# Community hospitals of the future—challenges and opportunities

**DOI:** 10.3389/frhs.2023.1168429

**Published:** 2023-08-09

**Authors:** Sean Kia Ann Phang, Ming Lin, Yong Xiang Kho, Rui Jie Rachel Toh, Ting Ting Kuah, Yi Feng Lai, JiaJing Kim Xie

**Affiliations:** ^1^ Nanyang Technological University, Singapore, Singapore; ^2^ Chua Thian Poh Community Leadership Centre, National University of Singapore, Singapore, Singapore; ^3^ MOH Office for Healthcare Transformation, Singapore, Singapore

**Keywords:** aging, healthcare, hospital, innovation, environmental scan

## Abstract

**Background:**

Medical training through specialization and even subspecialization has contributed significantly to clinical excellence in treating single acute conditions. However, the needs of complex patients go beyond single diseases, and there is a need to identify a group of generalists who are able to deliver cost-effective, holistic care to patients with multiple comorbidities and multi-faceted needs. Community hospitals (CHs) are a critical part of Singapore's shift toward a community-centric care model as the population ages. Community Hospitals of the Future (“CHoF”) represent a series of emerging conversations around approaches to reimagine and redesign care delivery in a CH setting in response to changing care needs.

**Methods:**

An environmental scan in the CH landscape using semi-structured interviews was conducted with 26 senior management, management, and working-level staff from seven community hospitals in Singapore. This environmental scan aims to understand the current barriers and future opportunities for CHs; to guide how CHs would have to shift in terms of (i) care delivery and resourcing, (ii) information flow, and (iii) financing; and to conceptualize CHoF to meet the changing care needs in Singapore.

**Findings:**

The analysis of all transcripts revealed four broad sections of themes: (i) current care delivery in CHs, (ii) current challenges of CHs, (iii) future opportunities, and (iv) challenges in reimagining CHs. An emerging theme regarding the current key performance indicators used also surfaced. Resource limitations and financing structure of CH surfaced as limitations to expanding its capability. However, room for expansion of CH roles tapping on the current expertise were acknowledged and shared.

**Conclusion:**

With the current issues of (i) rapidly aging population, (ii) specialist-centric healthcare system, and (iii) fragmentation of care ecosystem, there is a need to further understand how CHoF can be modeled to better tackle them. Therefore, several important questions have been devised to land us in a microscopic view on how to develop CHoF in the right constructs. Demographic changes, patient segmentation, service and regulatory parameters, patient's perspective, care delivery, and financial levers (or lack of) are some of the categories that the interview questions looked into. Therefore, the data gathered would be used to guide and refine the concept of CHoF.

## 1. Introduction

Singapore, like many other developed countries, is facing a rapidly aging population, where it is projected that 25% of the population would be 65 years and older by 2030 (1). With increasing age, there is an associated increased susceptibility for multimorbidity, which is widely defined by the diagnosis of two or more chronic diseases in an individual (2). The increasing aging population and prevalence of multimorbidity would result in higher demand for healthcare services, specifically hospital utilization, with an associated increased risk of hospitalization and longer average length of stay (ALOS) (3). Hospitals are already starting to face a bed crunch, with public sector admissions of older patients increasing from 28.6% in 2006 to 33.4% in 2013, and longer hospital stays from 7.8 days in 2010 to 8.2 days in 2013 (1). Yet, delivery of hospital-based care remains largely disease-centric, as patients with multimorbidity would be under the care of different subspecialists to manage each of their conditions, resulting in fragmented care and inefficiencies in the system (4, 5). While the healthcare system was initially designed to cost-effectively manage episodic conditions, adopting this approach to manage aging and rising multimorbidity would drive up healthcare costs unsustainably (6, 7).

Integrating healthcare services to provide person-centered care is a complex endeavor influenced by various enablers and barriers (8). Extensive research has identified key factors that facilitate or hinder successful care integration across diverse settings. Enablers include effective communication systems, shared electronic health records, collaborative care models, and strong leadership (9). Conversely, barriers such as fragmented healthcare systems, lack of coordination, inadequate information sharing, and resistance to change pose significant challenges (10). To guide the implementation of integrated care, frameworks like the World Health Organization's Integrated Person-Centred Care Framework offer comprehensive approaches that emphasize aligning health systems, services, and individual needs (11). This paper aims to explore the enablers and barriers to care integration while examining the applicability of existing frameworks in promoting successful implementation and sustainable improvements in healthcare delivery.

With the vision of delivering efficient and coordinated care, Singapore's healthcare services were reorganized into three integrated regional health systems in 2017 (12). Each regional health system would be capable of providing different levels of healthcare services across the care continuum, and this included the acute hospitals (AHs), community hospitals (CHs), and polyclinic groups (13, 14). However, care across multiple institutions still requires transfers, which leads to delays, additional bed days, and gaps in information shared (15).

At the acute hospital level, an example of integrated care designed to avoid the need for multiple transfers between institutions and care teams was the Integrated General Hospital (IGH) model. It demonstrated that a single generalist-led, multidisciplinary care team was able to provide efficient care and optimize resources according to the patient's acuity level, resulting in a shorter length of stay without compromising on patient safety (13, 15). It also highlighted that generalist clinicians are well placed to provide for complex care needs that span across multiple conditions, and specialist care can be provided as needed through multidisciplinary teams (13, 16, 17). While a generalist-led, integrated approach has shown to be effective in reducing hospital usage in patients with chronic diseases, cost savings from appropriate resourcing of care are rarely realized as integrated models of care are usually concentrated within the acute hospital setting with higher hospitalization costs (13, 17–20). Community hospitals, on the other hand, have services to cater for subacute medical needs and are considered lower resourced with a lower average daily bill, positioning themselves as a prime candidate for integrated hospital care (21, 22).

CHs are intermediate care facilities originally designed to manage patients discharged from the acute hospitals with continued rehabilitation needs (21). There are currently nine CHs in Singapore. Generally, most standalone CHs are operated by voluntary welfare organizations (VWOs), and most CHs are physically co-located with the AHs. While the majority of services in the CHs remain centered around inpatient rehabilitation as most clinicians in CHs are family medicine (FM)–trained generalists, there is potential to tap onto generalist care in CHs for patients with multimorbidity (23). Demonstrated by the Integrated General Medicine pilot for selected patient groups, FM physicians were able to take over care safely and effectively from the acute hospital at an earlier time point (24).

However, there is a paucity of research regarding the barriers and facilitators for the implementation of integrated care models in CHs, as most research efforts have explored and evaluated such care models in the AH setting (13, 20). To guide the envisioned delivery of such a model of care in CHs—termed Community Hospitals of the Future (CHoF), this study aims to explore the challenges that CHs face, to uncover future opportunities for CHs that would guide the conceptualization of CHoF, and to shift CHs to meet the needs of an aging population and rising multimorbidity.

## 2. Methods

### 2.1. Research design

The research questions for this study were, what are the key challenges faced by CHs in meeting the needs of an aging population and rising multimorbidity, and what future opportunities can be identified to guide the conceptualization of CHoF?

To answer these research questions, this qualitative environmental scan employed an inductive thematic analysis of semi-structured interviews collected in two phases: (1) phase 1 was approved by the National University of Singapore (NUS) Chua Thian Poh Community Leadership Centre's Department Ethics Review Committee (DERC); and (2) phase 2 was an internal study by MOH Office of Healthcare Transformation (MOHT) and approved by the NUS Institutional Review Board (IRB).

The semi-structured interviews were conducted to gather participants’ in-depth responses on a range of topic domains and emerging topics (25). Amid the COVID-19 pandemic situation, a remote interview protocol was employed to ensure the safety of researchers and the participants who were recruited from the healthcare setting. As such, all semi-structured interviews were conducted on video web-conferencing software, Zoom.

### 2.2. Research participants and recruitment

Research participants hailed from seven community hospitals: (1) Ren Ci Community Hospital; (2) St. Andrew's Community Hospital; (3) St Luke's Hospital; (4) Jurong Community Hospital; (5) SingHealth Community Hospitals (SCH); (6) Yishun Community Hospital; and (7) Woodlands Health Campus. Outram Community Hospital, Sengkang Community Hospital, and Bright Vision Hospital were classified as part of SingHealth Community Hospitals. These three hospitals were deemed as one research site as they have a shared management with duties and responsibilities that traversed across all three hospitals. All community hospitals in Singapore, except Ang Mo Kio-Thye Hua Kwan Hospital, responded to either phase of the study (Table 1).

**Table 1:** Composition of community hospital types and localities.

	Number of hospitals
Type of hospital^a^	
Government restructured	3
Voluntary welfare organization	3
Locality of hospital^a^	
Co-located	4
Standalone	2

^a^ The three SingHealth Community Hospitals include a mix of both types and localities. As such, number of interviewees and hospitals do not include SingHealth Community Hospitals.

Participants were recruited through a purposive snowball sampling of MOHT's available contacts and through representatives on the hospital's webpages. Initial shortlisted contacts and representatives were senior leaders from the CHs, and subsequent referrals included other Heads of Department, middle management, and working-level personnel. Senior leaders and Heads of Department included top-level personnel such as Chief Executive Officers; middle management level included roles such as Medical Leads of Departments; and working-level personnel include staff such as physiotherapists and nurses. When contacted, participants could voluntarily choose to participate. The research team also followed up with the participants by sending out an initial online demographic survey on Qualtrics (Supplementary Appendix A).

The inclusion criteria for both phases were that participants had to be or will be working in the Intermediate and Long-Term Care (ILTC) sector, currently working or will be working in a CH in Singapore, and must consent to be audio-recorded. The exclusion criteria were participants who were not able to speak English or who were unwilling to be audio-recorded. A total of 26 participants were interviewed (Table 2).

**Table 2:** Personnel interviewed.

	Number of interviewees
Community hospitals	
SingHealth Community Hospital^a^	9
Yishun Community Hospital	4
St. Luke's Hospital	4
St. Andrew Community Hospital	4
Woodlands Health Campus	2
Renci Community Hospital	2
Jurong Community Hospital	1
Type of hospital^b^	
Government restructured	7
Voluntary welfare organization	10
Locality of hospital^b^	
Co-located	11
Standalone	6
Profession	
Doctor	16
Operations	3
Allied health	3
Nursing	2
Finance	2
Position	
Heads of Departments	12
Senior management	8
Management	5
Working level	1

^a^ SingHealth Community Hospital is one research site comprising of three community hospitals that have a shared management.

^b^ The three SingHealth Community Hospitals include a mix of both types and both localities. As such, interviewees (*n* = 9) are not included in the table.

### 2.3. Data collection

The semi-structured interviews were conducted from February 2022 to June 2022. Each interview lasted between 1 and 2 h. There were two main segments to the interview (Supplementary Appendix B). First, participants were asked about the current situation in their respective CHs. This usually included open-ended questions about patient profiles and their current workflows with their attached acute hospitals.

In the second half of the interview, alternative CH models by MOHT (Supplementary Appendix B) were introduced and interviewee's responses were gathered. Key paradigm shifts proposed in these proposals included (a) the possibility of the community hospital being a gatekeeper to acute hospital services; (b) the increase in resources for community hospitals to take on a wider spectrum of patient acuity; and (c) an additional admission pathway from general practitioner (GP) clinics or emergency departments (EDs) directly into the community hospitals without having to first go through the acute hospital.

Audio recordings from Zoom were uploaded into the web-based artificial intelligence transcription software, Trint (26). The transcripts generated were vetted by researchers for accuracy.

### 2.4. Data analysis

In phase 1, thematic analysis was conducted on the 16 transcripts uploaded onto the Atlas.ti software. A reflexive approach to thematic analysis was eventually adopted to capture codes as they emerged from the transcripts (27). The thematic analysis included the following steps: (1) familiarize with the transcripts; (2) generate codes by attaching labels of meaning to parts of the transcript, with each transcript coded by two interviewers; (3) constructing themes, by reaching consensus in face-to-face discussions, about the central organizing ideas that capture coherent patterns of emerging codes across the dataset that answered the research question; (4) reviewing themes to ensure codes fit, were coherent, and distinct; and (5) clearly defining and naming themes (27, 28).

In phase 2, thematic analysis on the 10 transcripts was conducted by a codebook approach (27). In this analysis, interviewers created a codebook of codes and themes based on two transcripts. The codebook was refined by reaching consensus in face-to-face discussions. All 10 transcripts from phase 2 were then coded based on the codebook.

The findings were synthesized by merging matching themes from phase 1 and phase 2. For themes that did not align, a bigger theme was constructed to accommodate them.

## 3. Results

From the semi-structured interviews conducted, five main themes emerged. The results included interviewees’ insights into the current care delivery in CHs, current challenges of CHs, future opportunities, challenges in reimagining CHs, and an emerging theme regarding the current key performance indicators (KPIs) used.

### 3.1. Current care delivery in CHs

#### 3.1.1. Current patient profile and CHs role as an intermediary

The interviewees shared that the patient profile in CHs is currently made up of 70% rehab and 30% subacute patients, with the majority being elderly patients. This includes patients with dementia, chronic illness, mental health problems, neurodegenerative disorders, and patients with palliative care needs, as mentioned by ID08. With more CH patients having multimorbidity and social needs, ID15 referred to the current CH patient profile as “complex.”

Many interviewees talked about CHs’ role as an intermediary in the healthcare ecosystem and in terms of the services they provide. ID07 said that “the role of the community hospital is currently a step-down facility, intermediate facility, whereby we help patients who are not quite ready to go home yet to rehabilitate and regain their function as much as possible so that they can return back to their lives meaningfully as far as possible.” CHs also provide “engagement of caregiver training, medical reconciliation, identifying the needs and community services the patients require” (ID01).

#### 3.1.2. Current integrations between AHs and CHs

Current integrations between AHs and CHs were also discussed, where CHs can “manage patients of higher acuity with greater complexity” due to their “close proximity and distance” to acute hospital services (ID05). The physical proximity between CH and AH facilitates patient transfers, where “the patient can be in the acute hospital within 15 min or even less” (ID05). ID01 also shared that they “can even get the specialists to come over to take a look at the patient if required.” As such, the access to AH services increases the capability of CHs to handle higher acuity cases.

Another form of integration mentioned is a single management team across both the AH and CH, which is an advantage in terms of the ability to share and allocate resources. ID02 shared that “Depending on the needs of both hospitals, whichever site is busier, I can start to pull resources from the other side … [it] allows me to reduce a lot of structure idle time, helps to improve efficiency.”

In addition, stakeholders mentioned that CHs act as an extension of AHs. ID22 shared that “sometimes the line [is] quite blur[red], in terms of what type of patients should be in [an] acute hospital, what type of patient in CH.” ID15 also mentioned that “over time, [community hospitals are] going to be an extension of the acute side! Because the pressure now is so overwhelming on the acute side.” To free up beds in AH, CH functions as a step-down care facility. ID23 stated that “patients [who] no longer need very hyper acute or acute care management, they can then step down to the neighboring community hospital (…) without having to choke up an acute hospital bed.”

In addition, CHs act as the link between AH and the community, supporting patients’ discharge and aging in the community. Services such as respite care and caregiver support are provided for patients. ID16 shared that those patients who “can't navigate to resources that they need to keep themselves healthy, (…) [community hospitals will] try to link them up with community partners,” thereby facilitating the reintegration of patients back into the community.

### 3.2. Current challenges of CHs

#### 3.2.1. Diverse roles

One of the challenges brought up was the diverse roles of CHs. This arises from the lack of a clear definition and understanding of roles, varying services across CHs, and differences in the sources of funding between CHs. ID01 shared that “the current scope [of CHs] is actually very wide (…) may need to refine some of this scope that a community hospital needs to take.” ID05 also stated that “different community hospitals are able to manage patients of different acuity (…) there isn't a standardization for the term CH in Singapore”, highlighting the varying services and capabilities across CHs. The problem of diverse roles of CHs is exacerbated by the differences in the type of CHs, where the sources of funding differ. VWO CHs derive the bulk of their funding from donations to provide the resourcing needed for infrastructure, as mentioned by ID08. For government-owned CHs, ID15 opined that “there are different pockets of funding that are going to the government-run CHs.” The differences in the level of funding, therefore, results in the observed differences in the capabilities among CHs.

#### 3.2.2. Inefficient use of resources

##### 3.2.2.1. Patient choice toward specialist care creating demand for AH

The inefficient use of resources is another challenge faced by CHs. ID02 mentioned that “Singaporeans tend to have this assurance (…), big problem, small problem must also go see the specialist,” and this demonstrates how patient choice tends to gravitate toward specialist care, creating demand for AH.

##### 3.2.2.2. Discrepancy in subsidies in AH and CH

Patients’ refusal to go to CHs is also caused by discrepancies in subsidies in AH and CH. ID11 mentioned “the MediShield Life insurance has got a requirement that you need to be admitted to the acute hospital first before you can step down to the community hospital.” MediShield Life is a basic health insurance plan accessible to all Singaporeans that offsets the cost of large hospital bills (29). This requirement that ID11 mentioned suggests that the current funding structure creates an additional stream of patients who do not require AH care, going into AHs and generating a bottleneck.

ID24 reported “sometimes patients may not want to come to CH, [they] just prefer to stay on in the acute side because it's the sort of flat subsidy (…) So some do their math you know and then see, ‘actually it might be more expensive’. And also, there is a cap to how much you can use from MediSave for your CH stay (…).” MediSave is a scheme for individuals to put aside savings to cover healthcare expenses (30). In addition, the discrepancies in subsidies also stem from the current framework for means-testing. ID24 shared that “our patients are means-tested, according to the household per capita income. But in the acute hospitals, they use their individual incomes for their means test, (…) So in a way, the acute hospitals’ patients do kind of enjoy higher subsidies at the acute hospitals rather than at the community hospitals.”

As ID21 stated, “we [the community hospitals] are not able to provide these advanced imaging scans to our patients and it impedes our ability to provide a more seamless care for our patients because they do not then have access to MediShield Life and MediSave if they are to incur these charges at the community hospital if these services are delivered, because of the way this [financing policy] has been structured,” and this results in a disruptive care pathway between co-located CH and AHs.

##### 3.2.2.3. Barriers to discharge patients

With patients’ preference for AHs, and subsidy discrepancies that encourage AH stays, the increasing admissions and bed occupancy puts pressure on AHs to transfer patients to CHs. Due to the lower cost of beds in CHs, it is being seen as a decanting site. The increasing number of wrong-sited patients in CHs then results in poor use of resources such as beds and hinders the transfer of patients who would benefit from a CH stay. ID06 shared that many cases “may not have true rehab potential and are waiting for [nursing home] placement or [waiting to] sort out social issues, (…) but nonetheless they are still taking up beds in the CH.”

##### 3.2.2.4. Process challenges between AH and CH

The inefficient usage of resources also involves process challenges between CH and AH. Interviewees brought up the disruptive care pathways in co-located CHs, caused by the lack of funding for certain investigations. ID23 shared, “We can order, the CT here is going to cost $300 for the patient versus if we push the patient across to the acute hospital, and does it under the acute hospital stay, it is subsidized. So the same scan will cost a lot more in the community hospital.”

Some co-located CHs have certain processes arranged to help reduce the disruptions caused by the subsidy discrepancies. ID18 stated that “we create an outpatient visit and all that kind of things, some under the door method”; however, he says that it “just means this [current policy] gives more work for everybody on the ground.” Regulations that restrict CH doctors to order certain tests also contribute to the extra administrative work. ID26 mentioned that “by regulation, we (…) need to trouble acute hospital doctors, call them up, disturb them (…) to be very frank, I don't know whether that is productive use of time because you take up two doctors time (…), there's productivity loss.”

The lack of automated machines also causes processes in CHs to be relatively manual as compared to AHs, which translates to extra administrative work. ID24 shared that “acute sides, they have robotics to pack medications where they have these big robots right in the pharmacy (…) versus manual [in community hospitals] (…) because of funding, we can't have all that automated system [available] in the acute hospital.”

The unclear pathway when patients deteriorate in CH is also another source of process challenge between CH and AH. ID25 shared “so end up, the current [process] is, as long as you identify somebody that is not well, please go to the emergency room. Go to ER. Then, it defeats the purpose right? We are a hospital, you should trust our review that this patient requires a U-turn back. (…) And then when this patient goes back, want to U-turn to [the] acute hospital.”

ID20 shared that the AH physicians’ lack of ownership of patients after they are transferred to the CH affects patient care. “I think [it] all boils down to ownership (of the patient) (…). You feel that you’ve been called upon, right, to have a look, which was previously your patient, right?”.

##### 3.2.2.5. AH–CH-community partners

Links between the AH, CH, and community partners allows for partnerships to distribute patients across the facilities, sharing available resources. However, ID25 mentioned that insufficient hospital beds cause pressure to flow patients downstream as “AH bed status has been an issue, they are too crowded [and] not enough space so they tend to ask for help. So we started taking in patients much earlier that means before their acute issue is fully stable.”

However, ID18 also shared on the bottleneck experienced in the flow of patients from CHs to community care facilities such as nursing homes. “It's only that few centres around. And then if you have patients who need to go there, you need to basically wait for a bed. When there's no bed, there is no way that we can flow the patient down. So the pain point of this is the flow downstream when there are no physical beds, then even though you can collaborate, but no bed means no bed, patients can't flow, so [the] patient [is] stuck.” This leads to the inefficient use of resources despite collaborations between AH, CH, and community partners.

#### 3.2.3. Fragmentation of information sharing

Another challenge that CHs face is the fragmentation in data, leading to inefficiencies. ID08 shared that “you have to refer to multiple sources to really find out what's the background of the patient, if you hadn't been the doctor taking care of the patient before.” ID07 also mentions that “patients whom we do not have information on, a lot of time has to be spent back and forth through various systems, through paper, through faxing and all this can be cut down if there's a good system of information flow.”

Second, there is a fragmentation between care teams in the AHs and CHs. ID10 shared that “most times acute hospital and community hospitals are like silos, [patients] flow from one place to another place. And it's out of my care [acute perspective], then community hospital try to manage the case, but once it exceeds their capability or they don't know what to do, is to send the patient back to the acute side.”

Interviewees also brought up the need for better communication between AHs and CHs. ID25 said that the changing of junior doctors in AH affects communication. ID26 also shared on the current one-way communication from AH to CH. He said that “sometimes people don't provide enough information (…), we (the community hospital) can't do it or we need help, we can reach out but whether the other party is interested or not, yeah then think we can reach out but they’re not interested.”

Finally, the challenge of fragmentation also arises from the lack of a centralized IT system that involves CHs. ID13 mentioned that “a lot of the times community hospitals are not really, truly engaged, even in the design of the system.”

#### 3.2.4. Financing regulations

Problems with the current financing regulations is another challenge faced by CHs. Current subsidies encourage longer CH stays, as mentioned by ID06, “Patients do get a certain amount of payout for the days they are being hospitalized, this does not incentivize them to be discharged early.”

ID01 also shared that “some of these specialized scans are also not subsidized at the CH level, that's why then we also have to send back to the acute hospital, otherwise the payment from the patient is very high.” The lack of subsidies therefore results in higher out-of-pocket for patients in CHs.

The last challenge in terms of financing regulations is the source of financial support for VWO CHs. ID08 stated that VWO CH services are not within the scope of MOH goals and “whatever is out of alignment with MOH goals would then not have the needed or the needful funding.”

#### 3.2.5. Manpower and resources

The lack of funding translates to manpower limitations in CHs. ID01 said that “the reason we heard why they are not able to increase the manpower is because of funding.” Current perceptions held by healthcare workers toward the CHs also contributes to the manpower crunch, as ID08 mentioned that it is “very difficult to fund manpower because everyone doesn't want to join the community hospital, because that's the whole reason why they didn't do internal medicine to start off with.” One cited reason was the discrepancy in salaries between the hospitals. ID15 stated that “all CHs are struggling to find the nurses and doctors and the therapists when they see that acute (hospital) side salaries are so high.” The opportunities for career progression available in AHs and the lack of training opportunities in CHs also contributes to the difficulty in recruiting and retaining healthcare workers. ID14 shares that “in the acute care side, when there's a leadership track (…) development of any staff as you get promoted. (…) This is not quite present in the [CH] setting.” ID15 also mentioned that in CHs, “you cannot offer that width or depth of the research opportunities, or you cannot afford the generous conference leave or generous conference allowance.” These differences result in the perception that working in the CHs would have less incentives as compared to AH, leading to recruitment issues. To encapsulate this perception, ID23 stated that “is so sexy to work in acute hospitals, you use the latest robotics, you deal with the most acute of conditions. Let's face it, all of us become professionals because we want to do top end work right. So it is then how we can convince them that they may not need to always choose the top end all the time.”

With the changing patient profile in CHs, there is a need for staff upskilling. The increase in aging patients in CH means that “the skillset that is required to manage a person with dementia or even end of life is really quite different, (…) so current staff needs to be better trained and they have to undergo training in order to do this.” as mentioned by ID11. ID15 also shared that with the rise in subacute care patients in CH, they are “trying to train (their) staff to upskill because subacute care means they have infections, they need antibiotics. They have heart failure, they need to be on furosemide. (…) And as a rehab ward, we are not familiar with all those things.”

Finally, there is an increase in the CH complex patient profile, but current resource regulations do not meet the changing needs. ID26 mentioned that “there's just this natural barrier, because, again, the regulation is built on perhaps the older version where the patient care is not so complex.” With its limited resources, CHs are unable to expand their scope of services. ID07 stated that they do not have lab services. “(…) So if you want to manage acute conditions, it's difficult to do that.”

### 3.3. Future opportunities

Interviewees also shared on the future opportunities for CHs in terms of patient profile, scope and services, expertise, ownership of patients, the ecosystem, and workflow.

#### 3.3.1. Patient profile and resourcing

ID07 shared that “(CH) patients are getting more complex because they are having more comorbidities as they live longer. (…) Singapore's population ages, this will be the trend, there’ll be [an] increasing number of patients who will live longer but with more comorbidities, with probably cancers also occurring at the same time and they may need palliative care.” Interviewees also mentioned that resources such as increased funding and adoption of technology-based solutions would be required to cater to this increasingly complex patient profile. ID10 shared that “as we move more proximal towards the acute phase, the care needs, both medical and nursing will become higher. If funding is appropriate, right, more people can be employed to look after patients.”

#### 3.3.2. Expansion of scope and services

Interviewees suggested opportunities for CH to expand their scope and services in the future. ID06 stated that “there's definitely room for CHs to be able to support [a] more subacute level of care.” CHs could also introduce more social support to reduce readmissions. ID07 commented that “it's not just the medical, so it's also the social issues that patients experience (…), so they may also have psychological needs also. (…) the role of CH in that sense would be to prevent them from being readmitted again to the acute setting.” This suggests that CHs could take on a larger role in ensuring low readmission rates.

In addition, ID04 shared that CHs could expand their scope and “start to actually play a slightly more important diagnostic and triaging role.” CHs could also be responsible for ensuring continuity of care, as mentioned by ID08, “What I find very powerful is for there to be a link between inpatient care, outpatient clinic care and home care which is the link being what we call the continuity of care for the patient. (…) the understanding of the patient and the general holistic care for the patient will become a lot more coordinated and a lot more person-centered and personalized.”

#### 3.3.3. Expertise and training in CHs

##### 3.3.3.1. Increase in training and expertise to fulfill envisioned role

Next, opportunities for expertise and training in CHs were brought up by interviewees. ID01 talked about the need for a “close integration between acute hospital and community hospital, [where] some flow of the internal medicine, palliative, rehab specialists, geriatricians, into the CH sector is helpful because the patient profile has changed from the past,” thus suggesting an opportunity to increase the level of expertise in CHs to fulfill its envisioned role. To ensure the increase in the level of expertise, defined training pathways would be needed. ID08 said that “there is still not so clear [of a] career and training path for the community hospital doctors.” ID10 also shared that CH doctors would “need some form of community experience (…). The ability to lead and facilitate multi-disciplinary discussion is also one skill set they need to have. (…) So I think training is one important thing to facilitate the successful transformation of the hospital.”

##### 3.3.3.2. CH expertise for community care partners

Interviewees also commented on the expertise to be expanded to support community care partners. ID11 shared about the possibility for nursing homes to “work closer with community hospitals for the community hospital to support them, fund nursing homes to work closely with the community hospital to provide that care and where needed to, to shift the patient, from nursing homes to the community hospitals to be managed either for a short period and send them back (to the nursing homes) again.” There could also be “a mobile community hospital team to go to the nursing home to manage this group of patients and help them” (ID11). In addition, ID23 talked about the possibility for CHs to act as a “training ground for the community providers and partners. (…) provide training materials, resources, organize courses for these staff that work in the community setting.”

##### 3.3.3.3. Shared information system for seamless care transition

In addition, interviewees talked about how the sharing of information between AH, CH, and community partners could better facilitate seamless care transitions. ID23 mentioned “in [our hospital], is the shared EMR [Electronic Medical Record]. Whatever is in the acute hospital records we can read it. (…) gives us that ease and comfort of mind that we can read everything, and we can actually make the rest of the process a lot faster.” ID23 also described the need for information exchange at the national level. “There are a lot of missing gaps here and there, so if you can determine a very viable minimum dataset that will cater to the majority of the patients that are being transferred from setting to setting without a drop in clinical care, that will be good.”

#### 3.3.4. Shared ownership

Next, interviewees talked about the opportunity for shared ownership of patients within the healthcare system. Integration through shared accountability for patient care would be needed as ID09 mentioned that “it would be helpful, I think if within each individual healthcare system, they draw up some form of governance and share that accountability, meaning that the patients is still under the care of this entire organization.” To ensure accountability and the shared ownership of patients, ID01 stated that “hospital specialists still must maintain oversight. (…) although we can all be the arms and legs and community partners to help support that, but someone must have that oversight view.”

#### 3.3.5. Ecosystem

In terms of future opportunities in the wider ecosystem, interviewees brought up the possibility for CHs to play a triaging role in the community. ID05 suggested a “common ED [Emergency department] and triage centre so that patients don't have to decide where they’re going. They will be assigned into the CH or the AH, depending on the acuity.” ID10 also raised the opportunity to expand primary care capabilities as CH starts to cover more subacute patients.

Within the ecosystem, closer collaboration with nursing homes should also be explored. ID06 suggested that “because such patients [from nursing homes] are generally the older adults and they may be easily deconditioned (…). So in a CH there would be better opportunity for us to ensure that [patients] can receive a basic level of rehab, to ensure that they do not also become too deconditioned from that episode.” In addition, CH could collaborate with community partners to provide social prescribing and continue long-term outpatient treatment. ID05 shared that “it is important for CHs to be able to prescribe, to do social prescribing and to have strong social partners so that they can do the social prescription.”

#### 3.3.6. Workflow

Next, interviewees identified future opportunities in the workflow across care teams.

##### 3.3.6.1. Standardization of care process needed across care teams

Interviewees stated that there is currently a discrepancy between care processes across care teams, with AHs doing more tests than CHs. ID01 shared that “doctors or the medical teams or the clinical teams at all settings must agree that if it is that clinical presentation, it is best that patient stay here, this is how it should be managed, (…) Now you appear at acute hospital, they are very cautious, they do everything. So there seems to be a discrepancy.”

##### 3.3.6.2. Direct admission for community care partners

To improve workflow in the future, ID18 also suggested the possibility of direct admission for community care partners straight to the CH. He discussed the opportunity to streamline the current process by identifying “a certain small group of patients in the nursing home, those who require very intense palliative, more sub-acute kind of … that means the needs of that patient cannot be just managed in the nursing home itself, but not so cost-savvy to actually go to the ED and end up in the general hospital and then after that referred to the community hospital.”

##### 3.3.6.3. Telemedicine during transition from CH–home–community care

Another area of opportunity is during the transition from CH to community care centres. Due to the long wait for enrollment into outpatient settings, certain rehab patients are discharged home first from CH. However, ID24 said that “it's not ideal because patients may not do it [their prescribed exercises] properly.” Therefore, there is an opportunity to employ telemedicine during this transition from CH to home to community care. ID24 shared the use of tele-rehab as “an interim measure, so that at least you can monitor the patients during the exercises. Not quite the same as the physical supervision, but at least something, make sure that they are doing their exercises and doing it properly.”

##### 3.3.6.4. Need for tech equipment for rehab/patient care

The need for technological equipment for rehabilitation and patient care was also discussed. ID14 raised the example of gait training where a harness could be used to “take the weight off so that people can move their joints and exercise their muscles (…) there are many (…) robotic-enhanced technology that helps rehabilitation (…) That means, [with] one session you can do a lot more.” ID18 also talked about the potential for “innovations (…) that we can maybe reduce fall or even better, prevent fall.”

##### 3.3.6.5. Use of technology to minimize manpower strain

The overall manpower strain experienced in CHs can be minimized with the use of technology, as mentioned by ID25, “technology helps to take off manpower and time.” However, ID18 noted that “there's a thin line to thread because when you do that, you disengage the human factor.”

##### 3.3.6.6. Need for CH-related research

Next, interviewees brought up the need for CH-related research to be conducted. ID12 mentioned opportunities for setting-based research in terms of “how to reduce the U-turn rates within the CH? (…) And how to optimize transition of care back into the community from the CH.” There should also be more patient-based research which can help address current challenges. ID18 stated the need for CH to consider “what kind of ways that they can help in terms of patients of this kind of biopsychosocial profile (…) And is there something that we can look into that can address a piece that can break the vicious cycle?”.

##### 3.3.6.7. Necessary conditions for successful implementation

Finally, interviewees shared on the conditions that are necessary for successful implementation of changes within CHs. ID16 stated that “the first thing is the community hospitals need an innovative team. (…) A group of people, multi-disciplinary: doctors, nurses, allied health who can spend dedicated time to look at this.” ID17 talked about the importance in proving that the innovation “is beneficial to the patients, and it's cost effective.” ID16 also mentioned that the innovations need to be time saving and “easy to implement.”

### 3.4. Challenges raised in reimagining CH—CHoF

Interviewees raised challenges that they foresee in reimagining CH, in response to the CHoF hypothetical models that were presented.

#### 3.4.1. If CH is gatekeeper/first point of contact

First, with CHs as the gatekeeper and first point of contact, interviewees pointed out the lack of an ED. ID21 stated “where does the ED sits (…) a lot of the admissions come from A&E [Accident and Emergency Clinic].”

For CHs to be the first point of contact, one barrier that was identified was the lack of manpower. ID12 shared that it is “very difficult [for a direct admission from nursing home to CH], only got one house officer or one medical officer running the whole CH. How are they going to admit?”

#### 3.4.2. Skills and motivation of GPs to refer to CH

Next, the current skills and motivation of GPs to refer patients to CH is a challenge in reimagining CH. ID18 shared that “you need somebody who is trained and capable enough to understand that it's no longer just about sharing the medical condition. It's about whole [of] healthcare financing.” He stated that GPs need that insight to think if they can manage the case in the community, instead of always sending to the hospital which takes up a higher cost. ID20 also stated that “for the GP side, they must have a vested interest, meaning that you must make them accountable also.”

#### 3.4.3. Securing CH's future positioning

To secure CH's future positioning, interviewees shared that it is crucial to predict future generations’ healthcare needs. ID17 mentioned the need to “prepare healthcare to meet the generation's needs.” ID22 also said that “we don't know how long the other virus or will never come. But the infrastructure should be able to tweak and be ready,” emphasizing the need to strengthen CH's pandemic readiness.

### 3.5. Emerging themes

#### 3.5.1. Lack of appropriate key performance indicators for patient care in CHs

Through the series of interviews, an emerging theme regarding the lack of appropriate KPI for patient care in CH was identified. ID03 shared that “sometimes when you set [the KPI for a] certain timing [to track U-turns], it can be (…) dangerous as well. We do not know whether our ground staff will really try to hit the KPI and game the system or not. For example, the patient is so ill and there's just one two hours and then we will hit our KPI, and we try to (…) hold the patient for a while more before we transfer. I think that's very dangerous.” Therefore, it should be noted that some interviewees were concerned with the appropriateness of current KPIs in CHs.

## 4. Discussion

The aging population and rising multimorbidity is likely to continue the burden placed on acute hospitals today, and while CHs could play a key role in alleviating this pressure, it is unclear what the potential barriers are from achieving this envisioned role. From this qualitative study, the main challenges identified were, first, the financial regulations in CHs such as lower subsidies compared to the AH resulting in a higher out-of-pocket payment for patients and less financial support for VWO CHs. This has downstream effects as seen in the lack of standardization of services across CHs, a preference for AHs that pressures AHs to decant patients to the CHs resulting in poorly sited patients leading to inefficient use of beds, and a reliance on services in the AHs to bring down costs for the patient which creates process inefficiencies. The increased load on CHs is worsened by manpower limitations, due to a lack of incentives in recruiting or retaining staff, and a lag in upskilling of staff to meet the changing patient profile. Furthermore, fragmentation of care between the hospitals and reported inconsistencies in the information across systems exacerbates the inefficiencies in care delivery.

The challenges raised underline the limitations in the current policies that CHs are regulated under. CHs were originally designed with the intention to provide rehabilitation services for patients that generally require a longer period of inpatient stay within a lower-resourced setting (21). However, with an aging population driving higher bed occupancy rates and longer ALOS in the AHs, this creates a downward pressure on CHs to support AHs in supporting the increase in healthcare demands (1). As a result, CHs are seen to be caring for a changing patient profile that it may not be sufficiently designed to handle, in the most cost-effective manner.

The opportunities proposed by the interviewees in terms of how CHs could better provide for an aging population can be broadly classified into (i) expanding service capabilities and (ii) processes to enable continuity of care.

A consensus was that CHs were starting to care for a greater proportion of patients in the earlier stages of the care continuum, such as subacute patients or providing support for earlier rehabilitation. This can be better supported with an expansion of the expertise available in CHs through clearer training pathways and recruitment of relevant manpower. Where manpower is still lacking, respondents mentioned how technology could alleviate the manpower strain as well as bolster patient and rehab care. Such research into the implementation of technology would benefit from a dedicated interdisciplinary research team. Taken together, these suggestions would help sustain CHs’ role in taking on a greater portion of the patients from AHs and enable earlier transfers from AHs to CHs.

The emphasis on social prescribing can also be strengthened in CHs, which aims to improve the social factors that contribute to health, with the end goal of reducing these patients’ dependence on healthcare services (31). SCH started to provide social prescribing from 2019, and its implementation highlighted the need for new competencies and improved access to community resources (32). By enhancing efforts to facilitate patients’ reintegration into the community after discharge, it can translate into better wellbeing and health in the community.

At the heart of integrated care is shared patient ownership between care teams. By breaking down silos between the hospitals and creating shared accountability, patient needs can be met at the appropriate level of care and in a coordinated fashion. This can be supported by tighter information flows between teams and information systems across hospitals. Closer collaboration with primary care providers, nursing homes, and social service providers would be beneficial in facilitating patient transfers as well. Clinical pathways should be designed to facilitate patient transitions smoothly and efficiently along the care continuum, and ultimately facilitating right-siting of patients.

### 4.1. A potential CHoF model

**Figure 1:**
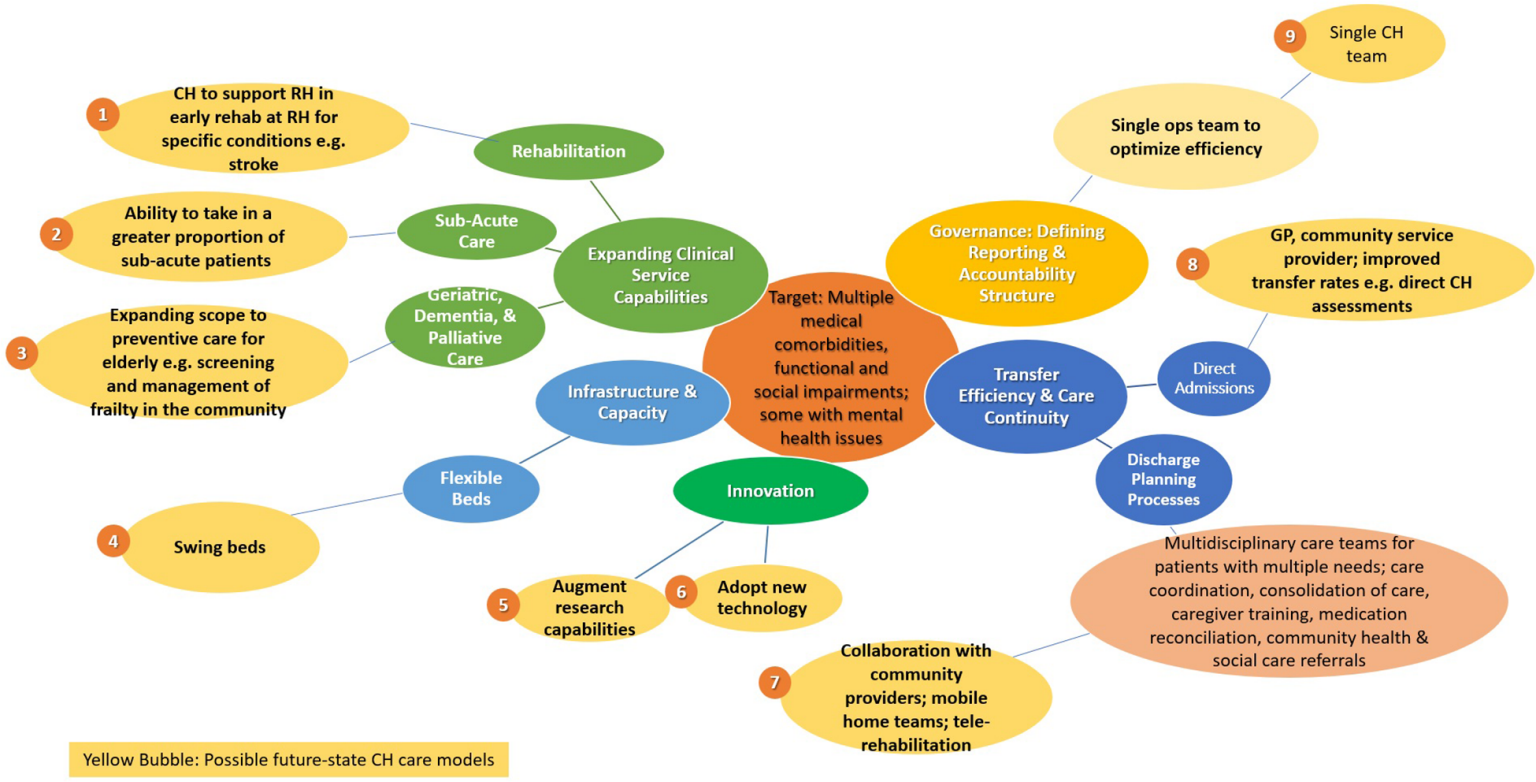
Key care redesign priorities from CHoF environmental scan.

An envisioned CHoF model would encompass non-specialist subacute care, with established clinical pathways for seamless transition of care between the AH, CH, and community partners. Figure 1 summarizes the key insights generated and the interpreted implications to care redesign priorities. Examples of such a care model could include (1) hospital in the community: home and community as the primary site of care by leveraging telemedicine, home/mobile care, and decentralization of services; (2) CH as a point of entry into the acute health system: CH as the default site of care for patients who may require a step-up from primary care, the point of entry/ gatekeeper for admission into the acute hospitals, and/or the site of elective surgical care; (3) early transfer pathways in and out of the CH: scaling across early transfer pathways into and out of the CH beyond disease-specific protocols, leveraging joint care with upstream and downstream providers, and providing outpatient continuing care for patients with needs beyond that of primary care; (4) ecosystems of care: CHs playing a bridging and coordinating role for health and social needs, hospital and community settings, and public and private providers within an ecosystem.

In the context of an aging population with rising multimorbidity, the CHoF model could provide cost-effective holistic care for patients who can be managed by a single multidisciplinary care team. However, reconceptualizing the care model for CHs cannot be done without accompanying changes in manpower and financing regulations, educating stakeholders in managing patients in a financially responsible manner, and perceptions toward the differences in quality of care patients may receive should be corrected for right-siting of care to happen.

### 4.2. Strength and limitations

As of the time of writing, this is the first qualitative study exploring the challenges and opportunities for CHs in Singapore. The data collected highlight prominent considerations for successful care transformation to happen in CHs. The use of semi-structured interviews allowed systematic gathering of data while providing flexibility for other information not covered in the interview guide to be gathered. Some limitations include the use of purposive snowball sampling in the recruitment of participants, which could have led to potential sampling bias. Since participants were selected based on referrals from initial participants, there was a risk of overrepresentation of certain characteristics, perspectives, or social networks within the sample. This could lead to a lack of diversity and the exclusion of individuals or groups who may have different or contrasting viewpoints, resulting in early data saturation and thus limited generalizability of the findings. In addition, the reliance on participant referrals may create a closed network, limiting the researcher's ability to access new or unique perspectives outside of the existing social connections. Future efforts to conceptualize CHoF should also include opinions from AH stakeholders and community partners, and factors that enable the scalability and sustainability of such a model should be explored as well.

## 5. Conclusion

With the current issues of a rapidly aging population, specialist-centric healthcare system, and fragmentation of care ecosystem, there is a need to further understand how CHoF can be modeled to better tackle these issues. Several important questions have been devised to land us in a microscopic view on how to develop CHoF in the right constructs. Demographic changes, patient segmentation, service and regulatory parameters, patient's perspective, care delivery, and financial levers (or lack of) are some of the categories that the interview questions explored. Therefore, the data gathered would be used to guide and refine the concept of CHoF. Moving forward, proof of concept (POC) is hoped to be demonstrated by running pilot programs at various sites and eventually implementing it at a larger scale. Considering the changing care needs arising from an aging population and increasing multimorbidity, future work would include exploring the factors to enable this concept to be scaled, sustained, and mainstreamed.

## Data Availability

The raw data supporting the conclusions of this article will be made available by the authors, without undue reservation.
