# Supplementary material for: Community hospitals of the future—challenges and opportunities
Source: Front Health Serv. 2023 Aug 9;3:1168429. doi: 10.3389/frhs.2023.1168429 (PMC10445538; doi:10.3389/frhs.2023.1168429)
Supplement: Supplementary file 1 [file Datasheet1.docx]

Supplementary Material

Community Hospitals of the Future (CHoF) – Challenges and Opportunities

Sean Kia Ann Phang^1^, Ming Lin^1^, Yong Xiang Kho^2^, Rui Jie Rachel Toh^2^, Ting Ting Kuah^2^, Yi Feng Lai^3*^, JiaJing Kim Xie^3^

* Correspondence: Lai Yi Feng: [yifeng.lai@moht.com.sg](mailto:yifeng.lai@moht.com.sg)

# Appendix A: Online Demographic Survey

Note: This form will be disseminated through Qualtrics.

1. Name
2. Are you currently above 21 years old?
3. Name of Community Hospital you are working in
4. What is your current job title in the Community Hospital?
5. How many years have you been working in the healthcare and community care sector?
   - Less than 1 year
   - 1 year
   - 2 years
   - Others (please specify)
6. How many years have you been working in a Community Hospital (CH)?
   - Less than 1 year
   - 1 year
   - 2 years
   - Others (please specify)
7. Could you briefly share your background? [E.g. previous titles, professions, specialties, years of practice, relevant experience]
8. On a scale of 1-5, how familiar are you with video conferencing platform, Zoom?
   - 1: Never used before
   - 2: Used once or twice, need help to set up
   - 3: Somewhat familiar, need advice.
   - 4: Familiar, only need little help setting up
   - 5: Very familiar, no help needed.
9. Do you consent to turning on your video during the Zoom session?
   - Yes
   - No
10. [Optional] If your response is 1-4, AND you would like us to help you to set up Zoom, please include your mobile contact details:

# Appendix B: Interview Guide

Current Situation

|  | **Questions** | **Optional Probes** |
| --- | --- | --- |
| Self-Introduction | Can you share what are your current roles and responsibilities? | (Based on their Qualtrics responses) |
| Patient-related Information  DRGs: Diagnosis Related Groups | **For HOD,**  What is your hospital’s current patient profile like? And how do you think the patient profile will change in years to come (acute/subacute inpatient subgroups/DRGs)?  **For Management/Working Level**  What are the patients you face most often?  And how do you think the patient profile will change in years to come (acute/subacute inpatient subgroups/DRGs)?  How will your work/workload or your role be impacted by change in the future? | Are there niche services that is unique to your Community Hospital? |

Opportunities & Challenges

|  | **Questions** | **Optional Probes** |
| --- | --- | --- |
| Hopes for the role of CH in Singapore Healthcare System | What roles do you hope to see CHs play in Singapore's healthcare system? | What are some health concerns that are not presently addressed that you think could be done in CHs? |
|  | How do you think future CHs can effectively provide holistic care for multimorbid patients? |  |
| Response to current policy | Collaborations with hospitals and community  How do you/your CH collaborate with acute hospitals now?  What do you think about the current collaboration with the acute hospital and community? And why?  What barriers are impeding collaborations among physicians? | If you are given a free hand without constraint, what changes do you hope to see in terms of collaborating with hospitals and the community?  What policies or regulations can be changed?  **For Family Medicine doctors/physicians/ HOD**, Do you think there are different roles between Internal Medicine (IM) specialist and Family Medicine (FM) physicians?  Is just having FM in CH enough or will other specialists be helpful?  Do you think the clinical expertise in CH is appropriate? What kind of changes in clinical expertise would be helpful?  → Possibility of having FM generalist and IM specialist working in a same setting   - In what aspect can FM physicians do differently? - How do you think IM in Acute Hospital can share care with FM at the community hospital? |
| Perception of Barriers faced for CHs  **Resources:**  **manpower**- staff to patient ratio, overall **subsidies/ funding for hospital**s, **medical equipment**)  **Costing**  Patient billing | What are some challenges your Community Hospital is facing right now?   - Among those, what may be the most critical problems?   What are the future challenges your Community Hospital is preparing for?   - Resources   - Manpower   - Costing (to patient groups) - **Patient selection:** Patient groups/Diagnostic Related Groups (DRGs) (e.g. stroke patients) [Medical capabilities] | What are some challenges your Community Hospital faces with regards to…  - Manpower  - Information flow (between different stages of care)  - CH’s facilities and resources (resourcing of care)  - Finances  - Policy  Do you have any feedback with regards to the current healthcare models and policies CHs are embedded in?   - Manpower - Policy - Communication   **For HOD/Management,**  Are there feedbacks that you hear from your staff and colleagues? What are they? |

Paradigm Shift/Feedback on the New Model

We are going to show possible new models of Community Hospitals and get your thoughts on it.

| Follow up from diagram | What do you think about this model?   - Benefits - Challenges   How should we measure success for this model? | Does it make sense?  Do you think it is possible that CH support “community-up” flows to avoid ED admissions (allowing earlier CH-level intervention before further exacerbation for defined patient segments)?  Why do you think so?  How do you think this would impact your workflow? And what would ease it?  What shifts would be required for the abovementioned model (*Section 4*) to work?  ***Diagram: what would it look like if they draw it (horizontal and vertical)*** |
| --- | --- | --- |
|  | **For FM doctors/physicians**, what roles can CHs and FM doctors/physicians play to meet the objectives of the abovementioned?  **For working level personnel,** how would you like to be supported in such changes of the potential model? | What do you think are the resources needed for the CH to take in more patients?  What are the expertise needed for this to happen?   - GPs - Doctors |
| Understand current limitations, barriers and facilitators of current regulatory policies of CHs in Singapore. | What are some potential ideas, or even policy changes, concerning Community Hospitals, that can be considered? | What financing/ care delivery and transition policies can MOH consider updating to improve care/acuity transition and promote better right-siting of care? |
|  | What are some of the further changes and improvements you hope to see?   - Care Redesign; Tech Insertion OR Job/Role Redesign   What are some potential ideas, or even policy enhancements, MOHT can consider? | How do you feel about the new Population Health spending? What effects would it have on you and your organisation? |
|  | What are your thoughts on existing expertise in CHs?  Why?  What are some expertise that you think is important for CHs but is lacking in the sector now? Why?   - Education - Discipline | What type of manpower plans should be in place (e.g. training)? |
